# Using machine learning to predict individual patient toxicities from cancer treatments

**DOI:** 10.1007/s00520-022-07156-6

**Published:** 2022-05-25

**Authors:** Katherine Marie Cole, Mark Clemons, Sharon McGee, Mashari Alzahrani, Gail Larocque, Fiona MacDonald, Michelle Liu, Gregory R. Pond, Lucy Mosquera, Lisa Vandermeer, Brian Hutton, Ardelle Piper, Ricardo Fernandes, Khaled El Emam

**Affiliations:** 1 Department of Medicine, Division of Medical Oncology, The University of Ottawa, Ottawa, Canada; 2 Cancer Therapeutics Program, The Ottawa Hospital Research Institute, Ottawa, ON Canada; 3 The Ottawa Hospital Cancer Centre, Ottawa, Canada; 4 Department of Oncology, McMaster University, Hamilton, ON Canada; 5 CHEO Research Institute, University of Ottawa, 401 Smyth Road, Ottawa, ON K1H 8L1 Canada; 6 Clinical Epidemiology Program, The Ottawa Hospital Research Institute, Ottawa, ON Canada; 7 University of Ottawa Health Services, Ottawa, ON Canada; 8 Division of Medical Oncology, Department of Oncology, Schulich School of Medicine & Dentistry, Western University, London, ON Canada; 9 School of Epidemiology and Public Health, University of Ottawa, Ottawa, ON Canada

**Keywords:** Vasomotor symptoms, Hot flashes, Breast cancer, Machine learning, Artificial intelligence, Survivorship

## Abstract

**Purpose:**

Machine learning (ML) is a powerful tool for interrogating datasets and learning relationships between multiple variables. We utilized a ML model to identify those early breast cancer (EBC) patients at highest risk of developing severe vasomotor symptoms (VMS).

**Methods:**

A gradient boosted decision model utilizing cross-sectional survey data from 360 EBC patients was created. Seventeen patient- and treatment-specific variables were considered in the model. The outcome variable was based on the Hot Flush Night Sweats (HFNS) Problem Rating Score, and individual scores were dichotomized around the median to indicate individuals with high and low problem scores. Model accuracy was assessed using the area under the receiver operating curve, and conditional partial dependence plots were constructed to illustrate relationships between variables and the outcome of interest.

**Results:**

The model area under the ROC curve was 0.731 (SD 0.074). The most important variables in the model were as follows: the number of hot flashes per week, age, the prescription, or use of drug interventions to manage VMS, whether patients were asked about VMS in routine follow-up visits, and the presence or absence of changes to breast cancer treatments due to VMS. A threshold of 17 hot flashes per week was identified as being more predictive of severe VMS. Patients between the ages of 49 and 63 were more likely to report severe symptoms.

**Conclusion:**

Machine learning is a unique tool for predicting severe VMS. The use of ML to assess other treatment-related toxicities and their management requires further study.

**Supplementary Information:**

The online version contains supplementary material available at 10.1007/s00520-022-07156-6.

## Introduction

Machine learning (ML) is an “application of artificial intelligence (AI) that allows computer systems to automatically learn from experience without explicit programming” [1]. Supervised learning models used in the oncology sphere, such as ensemble models (including gradient boosted trees) [2, 3], create predictions based on the aggregation of multiple individual models, and as such, can capture more complex relationships among variables [4]. In the setting of breast cancer, ML has been successfully applied for models examining risk of developing breast cancer [5], as well as breast cancer prognosis [6].

Vasomotor symptoms (VMS), including hot flashes and night sweats, are a common sequelae of systemic therapies for breast cancer [7, 8], and are the most common reason for discontinuation of potentially curative treatment [9–12]. As adjuvant therapies may be prescribed for up to 10 years [13, 14], appropriate management of VMS is essential to improve patient quality of life and breast cancer outcomes. Despite randomized trials showing effective interventions for the management of VMS [15], and recommendations from guideline groups that patients be routinely asked about VMS [16, 17], it is evident that VMS remain poorly managed [18, 19]. There are many reasons for this finding, including health care provider (HCP) uncertainty regarding non-pharmacologic strategies for VMS [20], the variability in frequency and severity of VMS in EBC patients [21], heterogeneity in the efficacy of interventions across the EBC population [15], and the absence of guidelines on tailoring treatments to individual patients.

Given the heterogenous nature of the breast cancer population, the variability in VMS severity, and the multiple interventions for VMS management, new strategies for implementing patient centered care are required. The objective of the current study was to create a gradient boosted decision tree (GBDT) model to identify factors that predict patients at risk of severe VMS as defined by the HFNS problem rating score.

## Materials and methods

### Survey dataset

We recently conducted a survey in patients with EBC who were experiencing VMS [22] (A.1). The objective of the survey was to determine patient perspectives on the frequency and severity of VMS, and to determine the effectiveness of previously tried interventions for this problem. After collecting demographic data including menopausal status and previous systemic therapies for breast cancer, patients rated the frequency and severity of their VMS using the validated Hot Flush Night Sweats (HFNS) Problem Rating Score [23]. This is a composite score that takes the mean of the problem, distress, and disruption to daily life caused by VMS. Each of the 3 variables is classified on a 10-point scale, with “1” representing low severity, and “10” representing significant severity. The final section of the survey asked patients to report on interventions that they had received for their VMS and to rate the effectiveness of these treatments. Patients were also asked how they themselves would define effective control of their VMS, and to provide feedback on specific types of interventions that they would be willing to try in the future.

### Data preparation

The outcome variable of interest was hot flash severity, as per the HFNS Problem Rating Score [23]. This outcome variable was chosen as it has been previously validated as a tool for assessing severity of VMS in the breast cancer population [24]. The three items in the HFNS score had a Cronbach alpha of 0.91 on our dataset, indicating high internal consistency [25]. In previous studies of women experiencing problematic or severe VMS, mean HFNS problem scores ranged from 5.88 to 6.3 (SD 2.2–2.6) [19, 24, 26]. Assuming that the distribution of HFNS problem scores follows a normal distribution, an integer cut-off score of 4 would capture approximately 84% of individuals with severe VMS (mean minus 1 standard deviation). A test of the normality assumption using the Kolmogorov–Smirnov (KS) statistic found *p* = 0.2263 (see Appendix C). Therefore, using this cut-off, we dichotomized patients into low and high severity scores. This coincides with the median, which results in a balanced dataset.

Variables were extracted from the patient survey questions for inclusion/exclusion in the ML model (Table B.1). Seventeen questions unrelated to the research question were excluded. These included one patient eligibility question, one patient feedback question, two questions relevant to patient treatment preferences, and four questions relevant to the perceived efficacy of interventions for VMS. To avoid redundancy, one additional question pertaining to menopausal status and two additional bothersome symptom questions were excluded. A question asking about previous treatments for VMS was removed as this was further explored in subsequent questions. Six questions relevant to hot flash severity were removed, including two questions pertaining to coping and control. While the two latter variables were part of the HFNS tool, these variables were found to be less reliable in the original validation study and were removed [23].

The remaining questions relevant to the analysis were as follows: patient age, previous systemic therapies for breast cancer, current menopausal status, hot flash/night sweats frequency, bothersome symptoms associated with VMS, changes made to breast cancer treatments due to VMS, recommendation of prescription or complementary and alternative medicine (CAM) interventions for VMS, and referral to a dedicated menopause clinic (Table 1). Responses to each question were assigned as binary or continuous variables. The most bothersome symptoms associated with VMS had 16 response options that were converted into binary variables. Variables that received less than 10% of valid responses were removed, which included 10 symptom variables (Table B.2). These variables had little variation, and therefore, were poor predictors of the outcome.

**Table 1:** Variables included in the creation of machine learning model

Variable	Definitions
1. Age**	Age in years
2. Menopausal status	Current self-reported menopausal status
3. Assessment of VMS	Whether the patient is asked about/assessed for symptoms of hot flashes by HCP during clinic visits
4. Hot flashes per week	The number of hot flashes in a week that occurred in the past week
Bothersome symptoms associated with VMS	Ranking of most bothersome symptom associated with VMS
5. Feeling extremely hot/sweaty	
6. Redness of my face/chest	
7. Feeling chills/clammy after hot flashes have passed	
8. Waking up at night/difficulty sleeping	
9. Irritability	
10. Memory problems	
11. Endocrine therapy	Endocrine therapy treatment for breast cancer (e.g., tamoxifen, letrozole/Femara, anastrozole/Arimidex)
12. Ovarian function suppression (OFS)	Ovarian function suppression treatment for breast cancer (leuprolide/Lupron, goserelin/Zoladex, oophorectomy)
13. Chemotherapy	Chemotherapy treatment for breast cancer
14. Change to BC treatment	Changes made to breast cancer treatment due to hot flashes
15. Drug treatments for VMS	Drugs that were prescribed or patient tried any prescription drug
16. CAM therapies for VMS	Complementary treatments prescribed, recommended, or tried by the patient
17. Referral to menopause clinic	Patient referred or seen by a gynecologist or dedicated menopause clinic to assist in managing hot flashes
Removed due to high correlation with “hot flashes per week” variable	
Hot flashes per day	The number of hot flashes per day that occurred in the past week
Nocturnal sweats per night	The number of times per night that nocturnal hot flashes (night sweats) woke you up in the last week
Nocturnal sweats per week	The number of times per week that nocturnal hot flashes (night sweats) woke you up in the last week

*VMS* = vasomotor symptoms, *HCP* = healthcare provider,  *BC* = Breast cancer, *CAM* = complementary and alternative medicine

^**^All variables converted to binary form with the exception of “age” and “number of VMS per day/week and night sweats per day/week”

### Analysis

The software used for analysis was R version 3.6, with main model construction using the *lightgbm* package (version 3.0.0). A GBDT was trained to predict the hot flash problem outcome [27, 28]. The basic process is to fit the data to a large number of trees, with each tree incorporating the prediction errors from the previous tree in a sequence as input. This approach has proven to have high prediction accuracy relative to linear models and deep learning models (see Appendix C), is robust in datasets that have missing values, and can model interactions and non-linear relationships without having them specified a priori. We used a nested cross-validation approach for model development and accuracy estimation (see Appendix C for further details). Bayesian optimization was used for hyperparameter selection [29]. Each combination of hyperparameters was evaluated using fivefold cross-validation on the training dataset during tuning (inner loop). Generalization accuracy scores were calculated using tenfold cross-validation whereby a trained model was used to predict the probability for unseen cases (outer loop).

The generalization accuracy of the predictions from the model was assessed using two metrics. The first was the area under the receiver operating characteristic curve (AUROC) [30], which is commonly used to evaluate the performance of binary classifiers in machine learning. It is a measure of the area under a plot of the false positive rate against recall. For binary classification tasks, an AUROC of 0.5 is the expected performance of a random classifier, while an AUROC of 1 is the expected performance of a perfect classifier. The second complementary metric which focuses on predictions of the positive class is the area under the precision-recall curve (AUPRC) [31]. The AUPRC value of a random classifier is the rate of the positive class [32] which is 0.5, with 1 being the perfect classifier.

### Model interpretation

We used two approaches to interpret the overall GBDT model: “permute and re-learn” to determine variable importance, and conditional partial dependence plots to visualize the functional form of the relationships between the predictors and the outcome.

Permutation of variables is a technique that involves the shuffling of variables to evaluate their impact on the accuracy of prediction models [33], and is commonly used to evaluate variable importance in machine learning models. There is evidence that permuting a variable is biased towards predictors that are correlated with other predictors that have many categories [34, 35]. Therefore, we instead permuted and reconstructed the model from the training data within each cross-validation iteration (permute and retrain the model), and then computed the difference in prediction accuracy between the original and permuted models [36, 37]. This difference gives the gain in accuracy by including a particular variable in the model. This will give us more reliable variable importance measures.

To illustrate the functional relationships between the most important predictor variables and the outcome of interest, conditional partial dependence plots were constructed [38]. Regular partial dependence plots are commonly used but have been subject to criticism as not all observations in the plot may plausibly be observed, leading to poor predictions due to extrapolation [37]. Conditional partial dependence plots aim to minimize extrapolation by calculating partial dependence within conditional subgroups, and then pools the results across subgroups. They also isolate the effect of a variable so we can view its impact, within the model, on the outcome.

## Results

### Description of the data

The original survey dataset comprised 383 patients. Ten patients were excluded, as these individuals were not experiencing VMS at the time of survey completion, totalling 373 patients who fulfilled eligibility (Table 2). An additional 13 patients were excluded from the gradient boosted trees analysis, as incomplete data was available for the HFNS problem rating score, totalling 360 patients included in the final analysis. These patients were recruited from the Ottawa Hospital Cancer Centre and the London Regional Cancer Centre, Ontario, Canada. As responses to all questions were optional, some variables had fewer than 360 responses.

**Table 2:** Baseline characteristics and summary statistics for patient sample, excluding symptom variables

Age	Number of respondents	Mean ± SD
Mean age	360*	56.3 ± 10.5
		***N* (%)**
18–24		1 (0.3%)
25–39		23 (6.4%)
40–59		204 (56.7%)
60–74		117 (32.5%)
75 +		15 (4.2%)
	Number of respondents	Median (IQR)
Hot flashes per week	295**	15 (IQR 5–35)
Binary variables included in the model		
	Yes or 1 count (% of total sample)	No or 0 count (% of total sample)
Menopausal at time of survey completion**	198 (55.0%)	130 (36.1%)
Routinely asked about VMS in clinic**	210 (58.3%)	129 (35.8%)
Endocrine therapy	319 (88.6%)	41 (11.4%)
Chemotherapy	205 (56.9%)	155 (43.1%)
Ovarian suppression	70 (19.4%)	290 (80.6%)
Change to BC treatment secondary to VMS	66 (18.3%)	294 (81.7%)
Drug treatments for VMS^¶^	112 (31.1%)	248 (68.9%)
CAM treatments for VMS^¶^	62 (17.2%)	298 (82.8%)
Referral to menopause clinic**^¶^	24 (6.7%)	335 (93.1%)

^*^Sample size fewer than 373 patients, as “I don’t know” and missing values were not counted

^**^Responses not provided by all survey participants

^¶^Included patients responding “yes” to any question containing this variable as an option

The mean age of the participants was 56.3 (SD 10.5) (Table 2). The majority of women were post-menopausal at the time of survey completion, and treatment received included endocrine therapy (*n* = 319/360, 88.6%) and chemotherapy (*n* = 205/360, 56.9%). A minority of patients reported receiving drug interventions (*n* = 112/248, 31.1%) or CAM interventions (*n* = 62/298, 17.2%) to manage their VMS, and 18% of patients reported changes in their breast cancer therapy secondary to VMS.

**Fig. 1:**
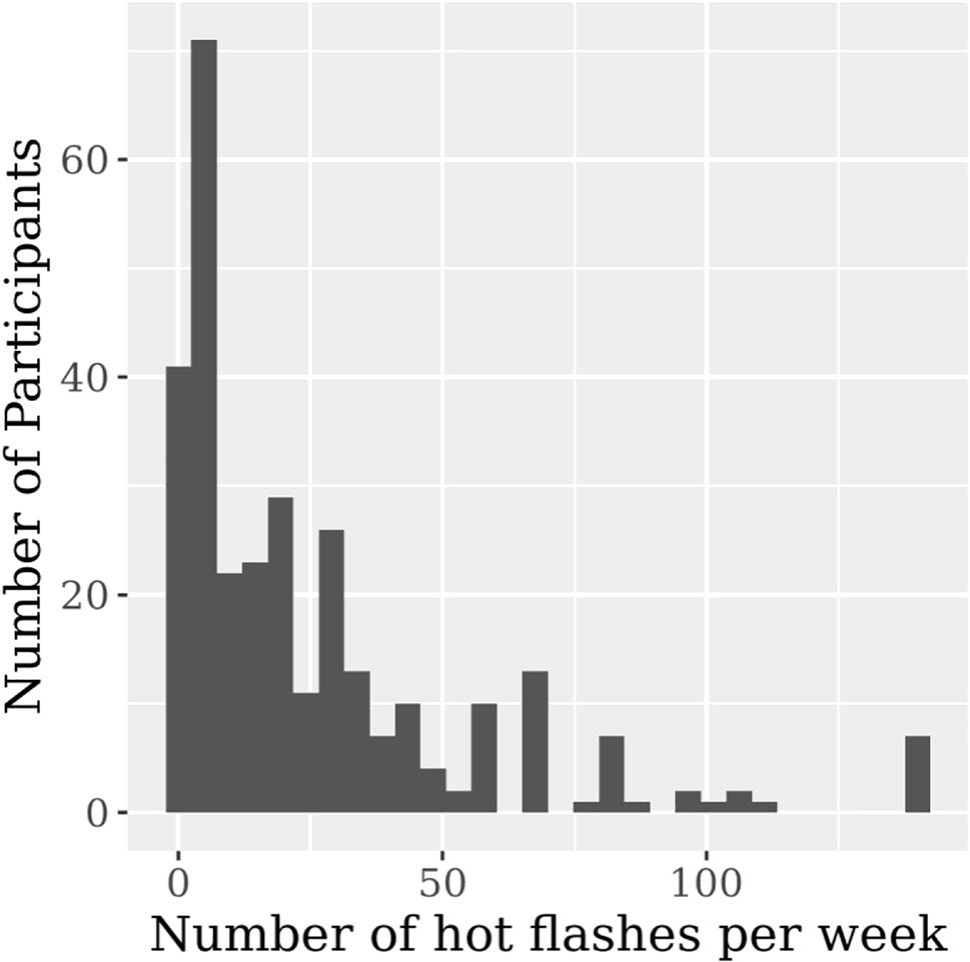
Distribution of the number of vasomotor symptoms per week. For this variable, counts at 140 hot flashes per week were topcoded, as there were very few observations above that threshold, and thus represented outliers in the population. This was done for the purposes of data presentation only, and affected a total of five patients (5/295, 1.7%)

The four variables pertaining to frequency of hot flashes and night sweats (hot flashes per day, hot flashes per week, night sweats per day, night sweats per week) were highly correlated (*r* > 0.7) (Table B.3). To avoid redundancy in the features that were used in modelling, we utilized the “hot flashes per week” variable only. The number of hot flashes per week is a commonly utilized endpoint in clinical trials of VMS in breast cancer patients, leading to its selection as the variable of interest [39, 40]. The median number of hot flashes per week was 15 (IQR 5–35) (Table 2, Fig. 1).

### Model accuracy and variable importance

**Fig. 2:**
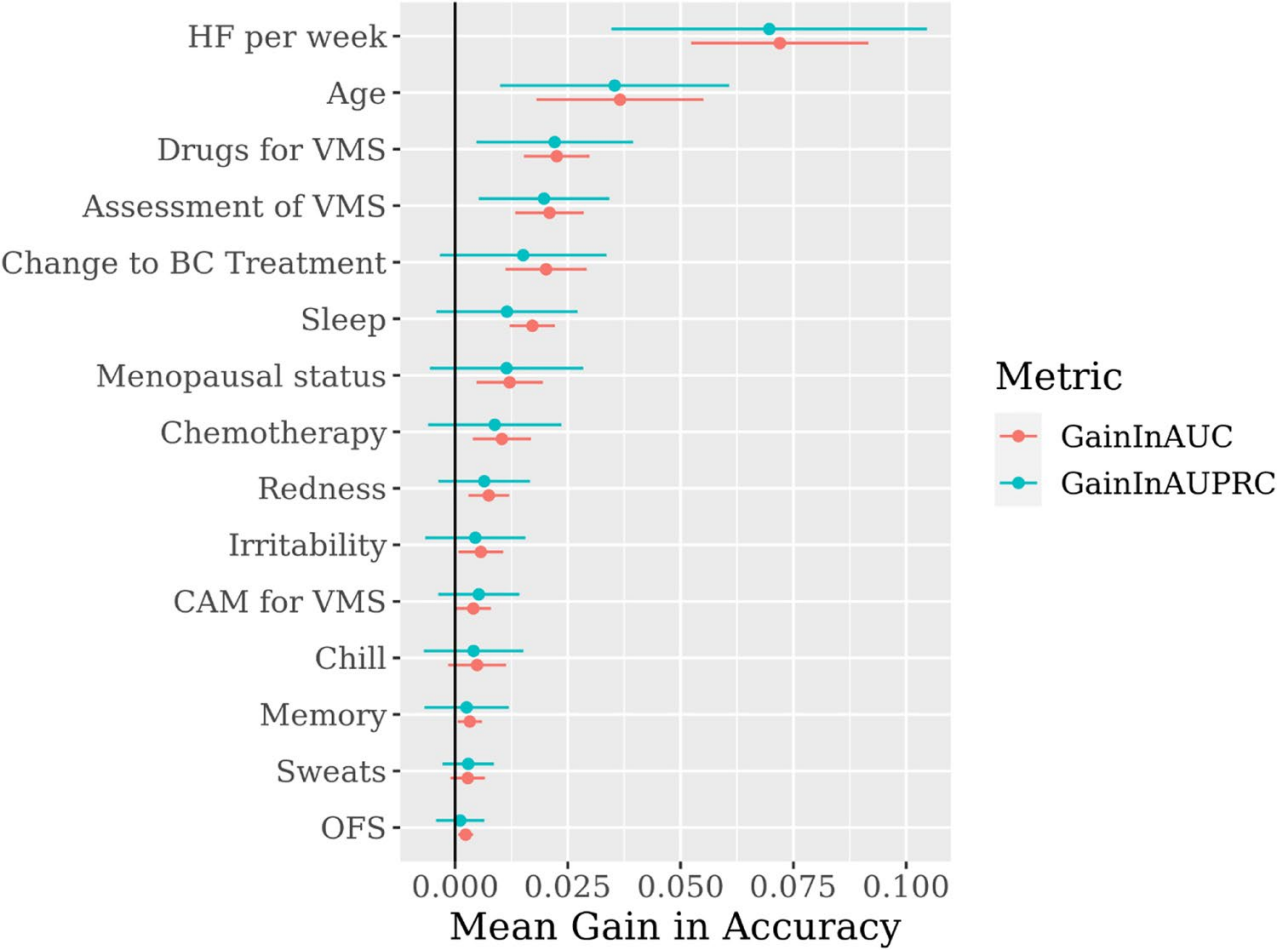
Variable importance using the permutation and retrain method with change in accuracy ± one standard deviation. The variables are ranked from the most important to the least important. “HF” is hot flashes, “VMS” is vasomotor symptoms, “BC” is breast cancer, “CAM” is complementary and alternative medicine, and “OFS” is ovarian function suppression

The model accuracy for predicting severity of VMS based on the AUROC value was 0.731 (SD ± 0.074) and the accuracy based on the AUPRC value was 0.687 (SD ± 0.079). The full confusion matrix and additional generalization performance metrics are included in Appendix C (Fig. C.3). The most important variable impacting the problem scale was the number of hot flashes per week (gain in AUROC 0.072 ± 0.019) (Fig. 2) followed by “age” (0.037 ± 0.018) and prescription and/or use of drugs to mitigate VMS (0.023 ± 0.007). Other highly ranked variables included whether the patient was asked about VMS in routine follow-up (0.021 ± 0.007) and whether changes were made to breast cancer treatment due to VMS (0.020 ± 0.009). The specific symptom variables had weaker impacts on the model, with waking up at night/difficulty sleeping (0.017 ± 0.005) having the greatest importance. Variables of lower importance included whether a patient had received or is still receiving endocrine therapy (0.0009 ± 0.0009), or ovarian function suppression treatments (0.0002 ± 0.001) for their breast cancer, which were the sixteenth and the fifteenth most important variables, respectively. The use of complementary medicines had lower relative importance in the model for the management of VMS (0.004 ± 0.004). The model with the six most important variables had similar generalization performance as the full model (see Appendix C, Table C.1). This suggests that a simpler model with fewer variables would provide comparable performance to the more complex model with 17 variables. We also removed the two variables with unclear causality (drug treatment for VMS and alteration of BC treatments) which gave poorer prediction performance than either of the other two models (Appendix C).

### Functional form of relationships

Conditional partial dependence plots were generated to show the functional form of the relationships between the most important variables and hot flash severity as per the HFNS Problem Rating Score. Each graph represents the impact of the variable factoring out the effects of the other variables. Given that the cut-off point for the outcome variable represented 50% of the observations, a predicted probability of a HFNS problem score of greater than 0.5 implies the presence of severe VMS.

**Fig. 3:**
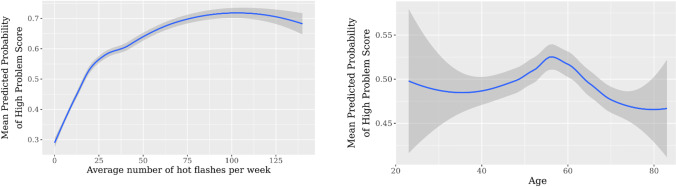
The probability of reporting severe vasomotor symptoms based on **a** the number of hot flashes per week and **b** patient age. The shaded region represents one standard deviation across the fivefold cross-validation

For the most important variable, which was the number of hot flashes per week, women who experience ≥ 17 hot flashes per week are more likely to consider their hot flashes as problematic (Fig. 3a). The peak probability score occurs at 103 hot flashes per week. Patients aged between 49 and 63 were more likely to report problematic hot flashes, with the maximum peak in women aged 56. Patients older than 63 were less likely to report problematic hot flashes (Fig. 3b). Among the 9 participants younger than or equal to age 35, 7 patients reported severe VMS (data not shown).

The probability of severe VMS was higher if a patient was offered a prescription or over-the-counter drug to manage their VMS (Fig. B.1a), and patients who reported that they were routinely asked about VMS were less likely to rate severe symptoms (Fig. B.1b). Finally, the probability of severe VMS was higher in patients who had a change in their breast cancer therapy due to VMS (Fig. B.2). The impact of the top two symptom variables that ranked of highest importance in the model was as follows: sleep disturbances and “redness of the face”/flushing (Fig. B.5). For both symptoms, individuals who reported experiencing sleep problems or “redness of the face/flushing” were estimated to have a higher probability of having problematic hot flashes.

## Discussion

Vasomotor symptoms are common in EBC patients [7, 8]; however, effectively managing this problem remains a challenge. The reasons for the complexity of this problem are many: unlike the general population, for example, estrogen replacement is contraindicated in breast cancer patients due to its role in tumorigenesis [41]. Moreover, a multitude of other challenges exist in managing VMS in this population, including systemic cancer treatments that worsen or induce VMS [7, 8], a lack of randomized controlled trials directly comparing active interventions for VMS [15], variation in patient preferences [19, 22], and health care provider uncertainty in the selection of optimal interventions for VMS [20].

While linear regression models serve as the backbone for traditional statistical modelling, they are limited in their capacity to capture complex interactions between variables, and as such, non-linear methods are often employed post hoc [42]. ML models can learn such complex relationships among multiple variables, making these techniques useful modalities for creating prediction models. Identifying patients at greatest risk of problematic and distressing VMS is critical to effective survivorship care in this patient population. In the current study using ML models, we have identified important factors that are predictive of patients at risk of severe VMS.

This is the first time, to our knowledge, that this approach has been applied to predicting patients at risk of severe VMS. Moreover, our results demonstrate that the frequency of hot flashes in a given week was the single greatest predictor of severe VMS as per the HFNS problem score. Our model has helped to identify that patients experiencing 17 or more hot flashes per week (or more than 2 hot flashes per day) are more likely to experience severe symptoms. This is an important finding, as it could be used in clinical practice to identify patients that can be offered early VMS interventions to help mitigate their symptoms. The results also demonstrate that women near the age of “natural menopause” (median age of 51 in Canada) [43] are more likely to report severe symptoms, which is consistent with previous studies in the non-breast cancer population demonstrating that severe VMS are more frequent in women transitioning to menopause or in early post-menopause, than in late menopause [44]. The importance of regular assessment of VMS in routine follow-up is emphasized by the finding that women who report being regularly asked about their symptoms are less likely to rate them as severe. This is an important finding, as only 58% of patients from our original patient survey reported being regularly asked about these symptoms in routine follow-up visits [22], which is substantiated elsewhere in the literature [19]. Similarly, while specific symptoms ranked lower in the model than the variables mentioned above, screening for sleep disturbances, which was the 6th most important variable, is an additional simple method to screen for patients at risk of distressing symptoms. Finally, while the recommendation for drugs to mitigate VMS, or changes to breast cancer treatments in response to poorly controlled VMS were associated with increased severity of symptoms, these variables, while interesting and important predictors (see Appendix C), do not assist in early detection of patients experiencing severe symptoms. This is an example of how a cross-sectional survey cannot disentangle cause and effect. Interestingly, exposure to endocrine therapy and ovarian function suppression rated as low importance in predicting distress from VMS, and will be evaluated further in a future prospective trial.

There are several limitations of this study. A study oversight involved the inclusion of an 11-point scale starting at “zero” included in the online version of the survey, and a 10-point scale starting at “one” in the paper version of the survey. While this oversight may introduce challenges comparing mean cumulative HFNS problem scores *between* patients, the dichotomization of high/low severity scores about the median (i.e., a score of 4) ensures that very low score individuals would be grouped together in the “low” problem score group, regardless of whether they utilized a scale beginning at “zero” or “one.” Moreover, the majority of patients conducted the survey electronically (259 electronic vs 114 paper), and thus utilized the 11-point scale. Secondly, while the survey from which the dataset was derived was conducted at a single-time point, the cross-sectional nature of the study does not permit evaluation of change in symptoms over time. As indicated above, the presence or absence of a drug treatment for VMS ranked highly in the variables of importance. This data indicates that patients with severe symptoms likely required escalated therapy; however, the ability of ML to predict efficacy of these medications remains unknown and requires a future prospective study to further assess this relationship. Recall bias also likely influences the results of this study, as patients were required to provide averages for the number of VMS per week, which can be subject to inaccuracies. Our survey did not ask patients about the severity of hot flashes prior to the breast cancer diagnosis, nor the age at diagnosis of menopause. These variables are likely to influence the problematic nature of VMS, and will be examined more closely in subsequent studies. The survey moreover did not integrate other risk factors for menopause, such as smoking, education, and socioeconomic status. Finally, the number of patients younger than 35, and those who utilized complementary therapies are small, and as such, their importance in the model will require further study.

Future prospective studies are needed to identify and treat patients experiencing bothersome VMS. We plan to use the above techniques in future trials to create prediction models that will guide patients and clinicians in the selection of individualized treatments for VMS, as well as the assessment of toxicities from other therapies.

## Conclusion

Machine learning offers a novel way to assess treatment toxicity in early breast cancer patients. In the current study, we demonstrated that the number of hot flashes per week and age were the two most important predictors for bothersome VMS in breast cancer patients. Patients experiencing more than 17 hot flashes per week are more likely to experience bothersome symptoms, and represent a higher risk group that may benefit from therapeutic interventions. Women between the ages of 49 and 63 are also more likely to report bothersome symptoms. Future studies specifically looking at these high-risk groups are needed.

## Supplementary Information

Below is the link to the electronic supplementary material.

Supplementary file1 (PDF 215 KB)

Supplementary file2 (DOCX 249 KB)

Supplementary file3 (DOCX 103 KB)

## Data Availability

The dataset generated during and/or analyzed during the current study are available from the corresponding author on request with approval of the Ontario Cancer Research Ethics Board.

## Declarations

### Statement on the welfare of animals

This article does not contain any studies with animals performed by any of the authors.

## References

[1] Rashidi HH, Tran NK, Betts EV, Howell LP, Green R (2019). Artificial intelligence and machine learning in pathology: the present landscape of supervised methods. Acad Pathol.

[2] Lynch CM, Abdollahi B, Fuqua JD, de Carlo AR, Bartholomai JA, Balgemann RN (2017). Prediction of lung cancer patient survival via supervised machine learning classification techniques. Int J Med Inform.

[3] Deist TM, Dankers FJWM, Valdes G, Wijsman R, Hsu IC, Oberije C (2018). Machine learning algorithms for outcome prediction in (chemo)radiotherapy: an empirical comparison of classifiers. Med Phys.

[4] Lu W, Fu D, Kong X, Huang Z, Hwang M, Zhu Y (2020). FOLFOX treatment response prediction in metastatic or recurrent colorectal cancer patients via machine learning algorithms. Cancer Med.

[5] Nindrea RD, Aryandono T, Lazuardi L, Dwiprahasto I (2018). Diagnostic accuracy of different machine learning algorithms for breast cancer risk calculation: a meta-analysis. Asian Pac J Cancer Prev.

[6] Montazeri M, Beigzadeh A (2016). Machine learning models in breast cancer survival prediction. Technol Health Care.

[7] Howell A, Cuzick J, Baum M, Buzdar A, Dowsett M, Forbes JF (2005). Results of the ATAC (Arimidex, Tamoxifen, Alone or in Combination) trial after completion of 5 years’ adjuvant treatment for breast cancer. Lancet.

[8] Pagani O, Regan MM, Walley BA, Fleming GF, Colleoni M, Lang I (2014). Adjuvant exemestane with ovarian suppression in premenopausal breast cancer. N Engl J Med.

[9] Chirgwin JH, Giobbie-Hurder A, Coates AS, Price KN, Ejlertsen B, Debled M (2016). Treatment adherence and its impact on disease-free survival in the Breast International Group 1–98 trial of tamoxifen and letrozole, alone and in sequence. J Clin Oncol.

[10] Hershman DL, Kushi LH, Shao T, Buono D, Kershenbaum A, Tsai WY (2010). Early discontinuation and nonadherence to adjuvant hormonal therapy in a cohort of 8,769 early-stage breast cancer patients. J Clin Oncol.

[11] McCowan C, Shearer J, Donnan PT, Dewar JA, Crilly M, Thompson AM (2008). Cohort study examining tamoxifen adherence and its relationship to mortality in women with breast cancer. Br J Cancer.

[12] Yood MU, Owusu C, Buist DS, Geiger AM, Field TS, Thwin SS (2008). Mortality impact of less-than-standard therapy in older breast cancer patients. J Am Coll Surg.

[13] Davies C, Pan H, Godwin J, Gray R, Arriagada R, Raina V (2013). Long-term effects of continuing adjuvant tamoxifen to 10 years versus stopping at 5 years after diagnosis of oestrogen receptor-positive breast cancer: ATLAS, a randomised trial. Lancet.

[14] Goss PE (2007). Letrozole in the extended adjuvant setting: MA.17. Breast Cancer Res Treat..

[15] Hutton B, Hersi M, Cheng W, Pratt M, Barbeau P, Mazzarello S (2020). Comparing interventions for management of hot flashes in patients with breast and prostate cancer: a systematic review with meta-analyses. Oncol Nurs Forum.

[16] Runowicz CD, Leach CR, Henry NL, Henry KS, Mackey HT, Cowens-Alvarado RL (2016). American Cancer Society/American Society of Clinical Oncology breast cancer survivorship care guideline. CA Cancer J Clin.

[17] Cardoso F, Kyriakides S, Ohno S, Penault-Llorca F, Poortmans P, Rubio IT (2019). Early breast cancer: ESMO clinical practice guidelines for diagnosis, treatment and follow-up†. Ann Oncol.

[18] Peate M, Saunders C, Cohen P, Hickey M (2021). Who is managing menopausal symptoms, sexual problems, mood and sleep disturbance after breast cancer and is it working? Findings from a large community-based survey of breast cancer survivors. Breast Cancer Res Treat.

[19] Fenlon D, Morgan A, Khambaita P, Mistry P, Dunn J, Ah-See ML (2017). Management of hot flushes in UK breast cancer patients: clinician and patient perspectives. J Psychosom Obstet Gynaecol.

[20] Cole KM, Clemons M, Alzahrani M, Larocque G, MacDonald F, Vandermeer L (2021). Developing patient-centred strategies to optimize the management of vasomotor symptoms in breast cancer patients: a survey of health care providers. Breast Cancer Res Treat.

[21] Chang HY, Jotwani AC, Lai YH, Jensen MP, Syrjala KL, Fann JR (2016). Hot flashes in breast cancer survivors: frequency, severity and impact. Breast.

[22] Cole K, Clemons M, El Emam K, Larocque G, MacDonald F, Vandermeer L (2022). Vasomotor symptoms in early breast cancer-a “real world” exploration of the patient experience. Support Care Cancer.

[23] Hunter MS, Liao KL (1995). A psychological analysis of menopausal hot flushes. Br J Clin Psychol.

[24] Mann E, Smith MJ, Hellier J, Balabanovic JA, Hamed H, Grunfeld EA (2012). Cognitive behavioural treatment for women who have menopausal symptoms after breast cancer treatment (MENOS 1): a randomised controlled trial. Lancet Oncol.

[25] Nunnally J, Bernstein IH (1994). Psychometric theory.

[26] Ayers B, Hunter MS (2013). Health-related quality of life of women with menopausal hot flushes and night sweats. Climacteric.

[27] Bühlmann Peter, Torsten H (2007). Boosting algorithms: regularization, prediction and model fitting. Stat Sci.

[28] Ke G, Meng Q, Finley T, Want T, Chen W, Weidong M, et al. (2017) LightGBM: a highly efficient gradient boosting decision tree [abstract]. NIPS’ 17: Proceedings of the 31st International Conference on Neural Information Processing Systems. Curran Associates Inc., Red Hook, NY, USA, 3149–57.

[29] Snoek J, Larochelle H, Adams R (2012) Practical Bayesian optimization of machine learning algorithms [abstract]. Proceedings of the 25th International Conference on Neural Information Processing Systems-Volume 2. Curran Associates Inc., Red Hook, NY, USA, 2951–59

[30] Pepe MS (2004). The statistical evaluation of medical tests for classification and prediction.

[31] Davis J, Goadrich M (2006) The relationship between precision-recall and ROC curves. Proceedings of the 23rd International Conference on Machine Learning (ICML). 148:233–40

[32] Boyd K, Costa VS, Davis J, Page CD (2012) Unachievable region in precision-recall space and its effect on empirical evaluation. Proceedings of the 29th International Coference on International Conference on Machine Learning. Edinburgh, Scotland: Omnipress. p. 1619–26. PMC3858955 24350304

[33] Janitza S, Strobl C, Boulesteix AL (2013). An AUC-based permutation variable importance measure for random forests. BMC Bioinf.

[34] Strobl C, Boulesteix A-L, Zeileis A, Hothorn T (2007). Bias in random forest variable importance measures: illustrations, sources and a solution. BMC Bioinf.

[35] Nicodemus KK, Malley JD, Strobl C, Ziegler A (2010). The behaviour of random forest permutation-based variable importance measures under predictor correlation. BMC Bioinf.

[36] Mentch L, Hooker G (2016). Quantifying uncertainty in random forests via confidence intervals and hypothesis tests. J Mach Learn Res.

[37] Hooker G, Mentch L (2019) Please stop permutating features: an explanation and alternatives. ArXiv, abs/1905.03151

[38] Molnar C, Konig G, Bischl B, Casalicchio G (2020) Model-agnostic feature importance and effects with dependent features- a conditional subgroup approach. http://arxiv.org/abs/2006.04628

[39] Biglia N, Bounous VE, Susini T, Pecchio S, Sgro LG, Tuninetti V, et al. (2018) Duloxetine and escitalopram for hot flushes: efficacy and compliance in breast cancer survivors. Eur J Cancer Care (Engl) 27 10.1111/ecc.12484 26936232

[40] Loprinzi CL, Kugler JW, Barton DL, Dueck AC, Tschetter LK, Nelimark RA (2007). Phase III trial of gabapentin alone or in conjunction with an antidepressant in the management of hot flashes in women who have inadequate control with an antidepressant alone: NCCTG N03C5. J Clin Oncol.

[41] Clemons M, Goss P (2001). Estrogen and the risk of breast cancer. N Engl J Med.

[42] Bertsimas D, Wiberg H (2020). Machine learning in oncology: methods, applications, and challenges. JCO Clin Cancer Inform.

[43] Costanian C, McCague H, Tamim H (2018). Age at natural menopause and its associated factors in Canada: cross-sectional analyses from the Canadian Longitudinal Study on Aging. Menopause.

[44] Mitchell ES, Woods NF (2015). Hot flush severity during the menopausal transition and early postmenopause: beyond hormones. Climacteric.

