# Supplementary material for: Using machine learning to predict individual patient toxicities from cancer treatments
Source: Support Care Cancer. 2022 May 25;30(9):7397–406. doi: 10.1007/s00520-022-07156-6 (PMC9385785; doi:10.1007/s00520-022-07156-6)
Supplement: Supplementary file 1 — Supplementary file1 (PDF 215 KB) [file 520_2022_7156_MOESM1_ESM.pdf]

Appendix A.
Sample Patient Survey

Using Machine Learning to Predict Individual Patient Toxicities from Cancer Treatments

Katherine Marie Cole a, Mark Clemonsa,b, Sharon McGeea, Mashari Alzahrania, Gail Larocquec, Fiona MacDonaldc, Michelle Luib, Gregory R. Pondd, Lucy Mosquerae, Lisa Vandermeerb, Brian Huttonf, Ardelle Piperg, Ricardo Fernandesh, Khaled El Emam*e,i

a.) The University of Ottawa, Department of Medicine, Division of Medical Oncology. 75 Laurier Ave. E, Ottawa, ON, Canada. K1N6N5.
b.) The Ottawa Hospital Research Institute, Cancer Therapeutics Program, Ottawa, Ontario, Canada.
c.) The Ottawa Hospital Cancer Centre. 501 Smyth Road. Ottawa, Ontario, Canada. K1H8L6
d.) McMaster University, Department of Oncology, Hamilton, Ontario, Canada.
e.) CHEO Research Institute, University of Ottawa, Ottawa, Ontario, Canada
f.) The Ottawa Hospital Research Institute, Clinical Epidemiology Program, Ottawa, Ontario, Canada.
g.) University of Ottawa Health Services, Ottawa, Ontario, Canada.
h.) Division of Medical Oncology, Department of Oncology, Schulich School of Medicine & Dentistry, Western University, London, Ontario, Canada.
i.) University of Ottawa, School of Epidemiology and Public Health, University of Ottawa, Ottawa, Ontario, Canada.

Correspondence: Khaled El Emam

CHEO Research Institute, 401 Smyth Road, Ottawa, Ontario K1H 8L1
Fax:
Tel: 613-797-5412
Email: kelemam@cheo.on.ca

REaCT-Hot Flashes Survey for Patients

Developing patient-centred strategies to optimise the management of vasomotor symptoms in patients with breast cancer. A survey of patients and health care providers

Breast cancer is the most commonly diagnosed malignancy in women. Hot flashes, or vasomotor symptoms, are experienced by the majority of breast cancer patients, and can negatively affect sleep, mood, and sexual function. While there are a number of available treatments for hot flashes, there is not sufficient evidence to help patients and physicians select one therapy over another. There is also a lack of understanding as to how patients themselves would define the optimal management and treatment of hot flashes. Our study will survey early stage breast cancer patients who have experienced hot flashes, with the goal of understanding which symptoms are most important to patients, and how we should define optimal control of this problem. Our study will additionally survey health care providers who treat patients with breast cancer, with the goal of understanding the current prescribing practices for managing symptoms of hot flashes.

This survey should take approximately 5-10 minutes to complete. All responses are anonymous.
Thank you for your participation.

* Required

1. Since your diagnosis of breast cancer, have you experienced hot flashes?

   Note: “Hot flashes” or “hot flushes” are experienced as a sudden feeling of heat, usually starting in the face/chest, and then spreading to the rest of the body. *

   ◯ Yes

   ◯ No

2. What is your age? _________ *

   The value must be a number

3. Please check all treatments that you have previously received/are currently receiving for your breast cancer. *

- [ ] Chemotherapy
- [ ] Endocrine therapy (eg. tamoxifen, letrozole/Femara, anastrozole/Arimidex, exemestane/Aromasin)
- [ ] Ovarian function suppression (leuprolide/Lupron, goserelin/Zoladex, oophorectomy (removal of ovaries))
- [ ] I don’t know

4. Are you currently experiencing menopause?
Note: “menopause” is defined as the absence of menstrual cycles/periods for 12 months. *

- ○ Yes
- ○ No
- ○ I don't know

5. Did your menopause symptoms happen before or after initiating treatment for breast cancer? *

- ○ Before
- ○ Afterwards
- ○ I don’t know

6. During a typical visit in the clinic, are you generally asked about symptoms of hot flashes?
(i.e., are you asked about hot flashes in more than 50% of your visits?) *

- ◯ Yes
- ◯ No
- ◯ I don't know

In this next section, we will ask questions about your specific hot flash symptoms.

We will use the Hot Flush Rating Scale (Hunter and Liao 1995).

Please answer the following 4 questions depending on what best applies to you and your hot flash symptoms (number per day or number per week).

7. In the past week, please estimate the number of hot flashes per day

The value must be a number

8. In the past week, please estimate the number of hot flashes per week

The value must be a number

9. If you have nocturnal hot flashes (night sweats), please estimate the number of times that these symptoms wake you up per night.

The value must be a number

10. If you have nocturnal hot flashes (night sweats), please estimate the number of times that these symptoms wake you up per week.

The value must be a number

Subjective assessment of hot flashes.

For the questions below, please circle your response on a scale of 0-10.

11. To what extent do you regard your hot flashes/night sweats as a problem?

0	1	2	3	4	5	6	7	8	9	10

Not a problem at all — Very much a problem

12. How distressed do you feel by your hot flashes?

0	1	2	3	4	5	6	7	8	9	10

Not a distressed at all — Very distressed

13. To what extent do your hot flashes interfere with your daily routine?

0	1	2	3	4	5	6	7	8	9	10

Not at all — Very much

14. How well are you coping with your hot flashes?

0	1	2	3	4	5	6	7	8	9	10

Not at all — Very much

15. How much control do you have over your hot flashes?

0	1	2	3	4	5	6	7	8	9	10

No control at all — Very good control

Ranking of hot flash symptoms

Based on your experiences with hot flases, please choose your top 3 most bothersome symptoms with #1 being the most bothersome.

16. Please select your 1st most bothersome symptom. *

- ◯ Feeling extremely hot/sweating
- ◯ Redness of my face/chest
- ◯ Feeling chills/clammy after hot flash has passed
- ◯ Palpitations (heart racing/beating quickly)
- ◯ Nausea
- ◯ Feeling dizzy
- ◯ Headaches
- ◯ Feeling suffocated during the event
- ◯ Waking up at night/difficulty sleeping
- ◯ Irritability
- ◯ Anxiety
- ◯ Feeling depressed/low mood
- ◯ Feeling a lack of control
- ◯ Memory problems
- ◯ Intimacy problems
- ◯ Work disruption
- ◯ None
- ◯ Other

17. Please select your 2nd most bothersome symptom.

- ◯ Feeling extremely hot/sweating
- ◯ Redness of my face/chest
- ◯ Feeling chills/clammy after hot flash has passed
- ◯ Palpitations (heart racing/beating quickly)
- ◯ Nausea
- ◯ Feeling dizzy
- ◯ Headaches
- ◯ Feeling suffocated during the event
- ◯ Waking up at night/difficulty sleeping
- ◯ Irritability
- ◯ Anxiety
- ◯ Feeling depressed/low mood
- ◯ Feeling a lack of control
- ◯ Memory problems
- ◯ Intimacy problems
- ◯ Work disruption
- ◯ None
- ◯ ______ Other

18. Please select your 3rd most bothersome symptom.

- ○ Feeling extremely hot/sweating
- ○ Redness of my face/chest
- ○ Feeling chills/clammy after hot flash has passed
- ○ Palpitations (heart racing/beating quickly)
- ○ Nausea
- ○ Feeling dizzy
- ○ Headaches
- ○ Feeling suffocated during the event
- ○ Waking up at night/difficulty sleeping
- ○ Irritability
- ○ Anxiety
- ○ Feeling depressed/low mood
- ○ Feeling a lack of control
- ○ Memory problems
- ○ Intimacy problems
- ○ Work disruption
- ○ None
- ○ Other

19. What is more bothersome to you – the severity (intensity) of your hot flashes or the frequency (number per day)? *

- ◯ Severity
- ◯ Frequency
- ◯ They are equally bothersome
- ◯ I don't know

In the next section, we will ask about treatments for hot flashes:

20. Were any changes made to your breast cancer treatment because of hot flashes?
Note: “Breast cancer treatment” in this question refers to tamoxifen, aromatase inhibitors, and/or ovarian function suppression
(Check all that apply) *

- ☐ My breast cancer treatment was held temporarily
- ☐ My breast cancer treatment was discontinued or stopped
- ☐ My breast cancer treatment was changed to another agent (eg. tamoxifen changed to an aromatase inhibitor, change of aromatase inhibitors, etc.)
- ☐ The dose of my breast cancer treatment was changed
- ☐ No changes were made
- ☐ I don’t know

21. If changes were made to your breast cancer treatment because of hot flashes, did you experience changes to your hot flash symptoms? Check all that apply. *

- ☐ No, I did not experience changes
- ☐ Yes, I experienced fewer hot flashes and/or night sweats
- ☐ Yes, I experienced less severe hot flashes and/or night sweats
- ☐ I don’t know
- ☐ ________

Other

22. Have you received treatment for your hot flashes since your breast cancer diagnosis?

Note: This can include both drug treatments (prescription or over-the-counter), and complementary therapies (eg. counselling, acupuncture, exercise, relaxation, etc.). This does NOT include changes made to your breast cancer treatment indicated in the previous question. *

- ◯ Yes
- ◯ No
- ◯ I don't know

23. Have you been prescribed drugs (prescription or over the counter) for your hot flashes? (Check all that apply)

- ☐ Anti-depressants (eg. venlafaxine, paroxetine (Paxil), citalopram (Celexa), escitalopram (Cipralex), duloxetine (Cymbalta), sertraline (Zoloft))
- ☐ Gabapentin
- ☐ Clonidine
- ☐ Hormone therapy (estrogens and/or progesterones)
- ☐ Vitamin E
- ☐ Black Cohosh
- ☐ Soy/ Milk protein
- ☐ Melatonin
- ☐ Red clover
- ☐ Evening primrose oil
- ☐ ______

Other

24. Have you been prescribed/recommended complementary treatments for your hot flashes?
(Check all that apply)

- [ ] Acupuncture
- [ ] Relaxation therapy
- [ ] Exercise therapy/Yoga
- [ ] CBT (cognitive behavioral therapy) or counselling
- [ ] Hypnosis
- [ ] ____________

Other

25. Since being diagnosed with breast cancer, have you ever been referred to a gynecologist/dedicated menopause clinic to assist in managing your hot flashes?

- ○ Yes
- ○ No

26. In your opinion, a treatment would be effective in controlling your hot flash symptoms if?
Note: You may select more than one option.

- [ ] The treatment decreased the number of hot flashes you had in a given week
- [ ] The treatment decreased the severity of the hot flashes that you had in a given week
- [ ] The treatment prevented you from waking up at night with night sweats (i.e., improved sleep)
- [ ] The treatment helped your low mood/anxiety/irritability related to your hot flash symptoms
- [ ] None
- [ ] ____________

Other

27. Based on your answers to the previous question, please indicate which of the following drug and/or complementary therapies have adequately controlled or improved your hot flash symptoms.
Note: You can choose more than one response if they have worked for you.

- ☐ Anti-depressants (eg. venlafaxine, paroxetine (Paxil), citalopram (Celexa), escitalopram (Cipralex), duloxetine (Cymbalta), sertraline (Zoloft))
- ☐ Gabapentin
- ☐ Clonidine
- ☐ Hormone therapy (estrogens and/or progesterones)
- ☐ Vitamin E
- ☐ Black Cohosh
- ☐ Soy/ Milk protein
- ☐ Melatonin
- ☐ Red clover
- ☐ Evening primrose oil
- ☐ Acupuncture
- ☐ Relaxation therapy
- ☐ Exercise therapy/Yoga
- ☐ CBT (cognitive behavioral therapy) or counselling
- ☐ Hypnosis
- ☐ Referral to menopause clinic/gynecologist
- ☐ No treatments were effective or adequately controlled my symptoms
- ☐ I have never tried one of these treatments as lifestyle modifications have been sufficient
- ☐ ______________

Other

28. Based on your answers to the two previous questions, please indicate which of the following drug and/or complementary therapies did NOT adequately control or improve your hot flash symptoms?
Note: You can choose more than one response.

- [ ] Anti-depressants (eg. venlafaxine, paroxetine (Paxil), citalopram (Celexa), escitalopram (Cipralex), duloxetine (Cymbalta), sertraline (Zoloft))
- [ ] Gabapentin
- [ ] Clonidine
- [ ] Hormone therapy (estrogens and/or progesterones)
- [ ] Vitamin E
- [ ] Black Cohosh
- [ ] Soy/ Milk protein
- [ ] Melatonin
- [ ] Red clover
- [ ] Evening primrose oil
- [ ] Acupuncture
- [ ] Relaxation therapy
- [ ] Exercise therapy/Yoga
- [ ] CBT (cognitive behavioral therapy) or counselling
- [ ] Hypnosis
- [ ] Referral to menopause clinic/gynecologist
- [ ] No treatments were effective or adequately controlled my symptoms
- [ ] I have never tried one of these treatments as lifestyle modifications have been sufficient
- [ ] ____________________

Other

29. If given the option, would you rather:

- ◯ Take a prescription medication for hot flashes (eg. venlafaxine, gabapentin, clonidine, etc.)
- ◯ Take a vitamin or supplement for hot flashes (eg. black cohosh, melatonin, etc.)
- ◯ Try a complementary therapy such as counselling, relaxation therapy, exercise, etc.
- ◯ Be referred to a specialized menopause clinic
- ◯ Any option is fine if it improves my symptoms
- ◯ None of the above, my symptoms are managed with lifestyle modifications

30. This survey is about you. Are there any further suggestions or questions that we did not address in this survey? Please add your comments in the space below.

Survey created using Microsoft Forms
