# Supplementary material for: Using machine learning to predict individual patient toxicities from cancer treatments
Source: Support Care Cancer. 2022 May 25;30(9):7397–406. doi: 10.1007/s00520-022-07156-6 (PMC9385785; doi:10.1007/s00520-022-07156-6)
Supplement: Supplementary file 2 — Supplementary file2 (DOCX 249 KB) [file 520_2022_7156_MOESM2_ESM.docx]

**Appendix B**

**Using Machine Learning to Predict Individual Patient Toxicities from Cancer Treatments**

Katherine Marie Cole ^a^, Mark Clemons^a,b^, Sharon McGee^a^, Mashari Alzahrani^a^, Gail Larocque^c^, Fiona MacDonald^c^, Michelle Lui^b^, Gregory R. Pond^d^, Lucy Mosquera^e^, Lisa Vandermeer^b^, Brian Hutton^f^, Ardelle Piper^g^, Ricardo Fernandes^h^, Khaled El Emam*^e,i^

a.) The University of Ottawa, Department of Medicine, Division of Medical Oncology. 75 Laurier Ave. E, Ottawa, ON, Canada. K1N6N5.

b.) The Ottawa Hospital Research Institute, Cancer Therapeutics Program, Ottawa, Ontario, Canada.

c.) The Ottawa Hospital Cancer Centre. 501 Smyth Road. Ottawa, Ontario, Canada. K1H8L6

d.) McMaster University, Department of Oncology, Hamilton, Ontario, Canada.

e.) CHEO Research Institute, University of Ottawa, Ottawa, Ontario, Canada

f.) The Ottawa Hospital Research Institute, Clinical Epidemiology Program, Ottawa, Ontario, Canada.

g.) University of Ottawa Health Services, Ottawa, Ontario, Canada.

h.) Division of Medical Oncology, Department of Oncology, Schulich School of Medicine & Dentistry, Western University, London, Ontario, Canada.

i.) University of Ottawa, School of Epidemiology and Public Health, University of Ottawa, Ottawa, Ontario, Canada.

**Correspondence:** Khaled El Emam

CHEO Research Institute, 401 Smyth Road, Ottawa, Ontario K1H 8L1

Fax:

Tel: 613-797-5412

Email: [kelemam@cheo.on.ca](mailto:kelemam@cheo.on.ca)

**Table B. 1** **Variables extracted from Patient Survey Questions for Inclusion/Exclusion in Machine Learning Model.** Bold text indicates variable of interest

| **Inclusion** | **Exclusion** |
| --- | --- |
| **Patient factors:**   - What is your **age**? - Are you currently experiencing **menopause**? - In the past week, please estimate the number of **hot flashes per day and week** - In the past week, please estimate the number of **night sweats per day and week** - Please select your **1st most bothersome symptom.** | **Eligibility/feedback questions:**   - Since your diagnosis of breast cancer, have you **experienced hot flashes**? - Are there any **further suggestions** or questions that we did not address in this survey? |
| **Breast Cancer treatment factors:**   - Please check all treatments that you have previously received/are currently receiving for your breast cancer. Eg, **Chemotherapy, endocrine therapy, ovarian Function suppression** - Were any **changes made to your breast cancer treatment** because of hot flashes? | **Redundant questions:**   - Did your menopause symptoms happen **before or after initiating treatment for breast cancer**? - Please select your **2^nd^ most bothersome symptom** - Please select your **3^rd^ most bothersome symptoms** - Have you received **treatment for your hot flashes since your breast cancer diagnosis**? |
| **Breast cancer assessment:**   - During a typical visit in the clinic, are you generally **asked about symptoms of hot flashes?** | **Excluded as pertaining to dependent variable/outcome of interest**   - To what extent do you regard your hot flashes/night sweats as a **problem**? - How **distressed** do you feel by your hot flashes? - To what extent do your hot flashes **interfere** with your daily routine? - How well are you **coping** with your hot flashes? - How much **control** do you have over your hot flashes? - What is **more bothersome to you** – the severity (intensity) of your hot flashes or the frequency (number per day)? |
| **Interventions for VMS:**   - Have you been **prescribed drugs** (prescription or over the counter) **for your hot flashes?** - Have you been **prescribed/recommended complementary treatments for your hot flashes?** - Since being diagnosed with breast cancer, have you ever **been referred to a gynecologist/dedicated menopause clinic** to assist in managing your hot flashes? | **Questions relevant to effectiveness of treatment:**   - In your opinion, a **treatment would be effective in controlling your hot flash symptoms if**?   Note: You may select more than one option.   - Please indicate which of the following drug and/or complementary therapies **have adequately controlled or improved your hot flash symptoms**. - Please indicate which of the following drug and/or complementary therapies **have not adequately controlled or improved your hot flash symptoms**. - If **changes were made to your breast cancer treatment because of hot flashes, did you experience changes to your hot flash symptoms?** |
|  | **Interest in future treatment**   - If given the option, **would you rather take a prescription medication, vitamin, CAM therapy, etc**? |

**Table B.2- Symptom variables with fewer than 10% valid responses**

| Work disruption | Intimacy problems |
| --- | --- |
| Nausea | Headaches |
| Feeling a lack of control | Feeling suffocated |
| Dizziness | Depression/low mood |
| Anxiety | Palpitations |

**Table B.3.** Correlation matrix (Pearson correlations) among the different measures of frequency of hot flashes.

|  | **Hot_Day** | **Hot_Week** | **Nocturnal_Day** | **Nocturnal_Week** |
| --- | --- | --- | --- | --- |
| **Hot_Day** | 1 | 0.984740 | 0.709090 | 0.799030 |
| **Hot_Week** | 0.984740 | 1 | 0.722895 | 0.815971 |
| **Nocturnal_Day** | 0.709090 | 0.722895 | 1 | 0.903582 |
| **Nocturnal_Week** | 0.799030 | 0.815971 | 0.903582 | 1 |


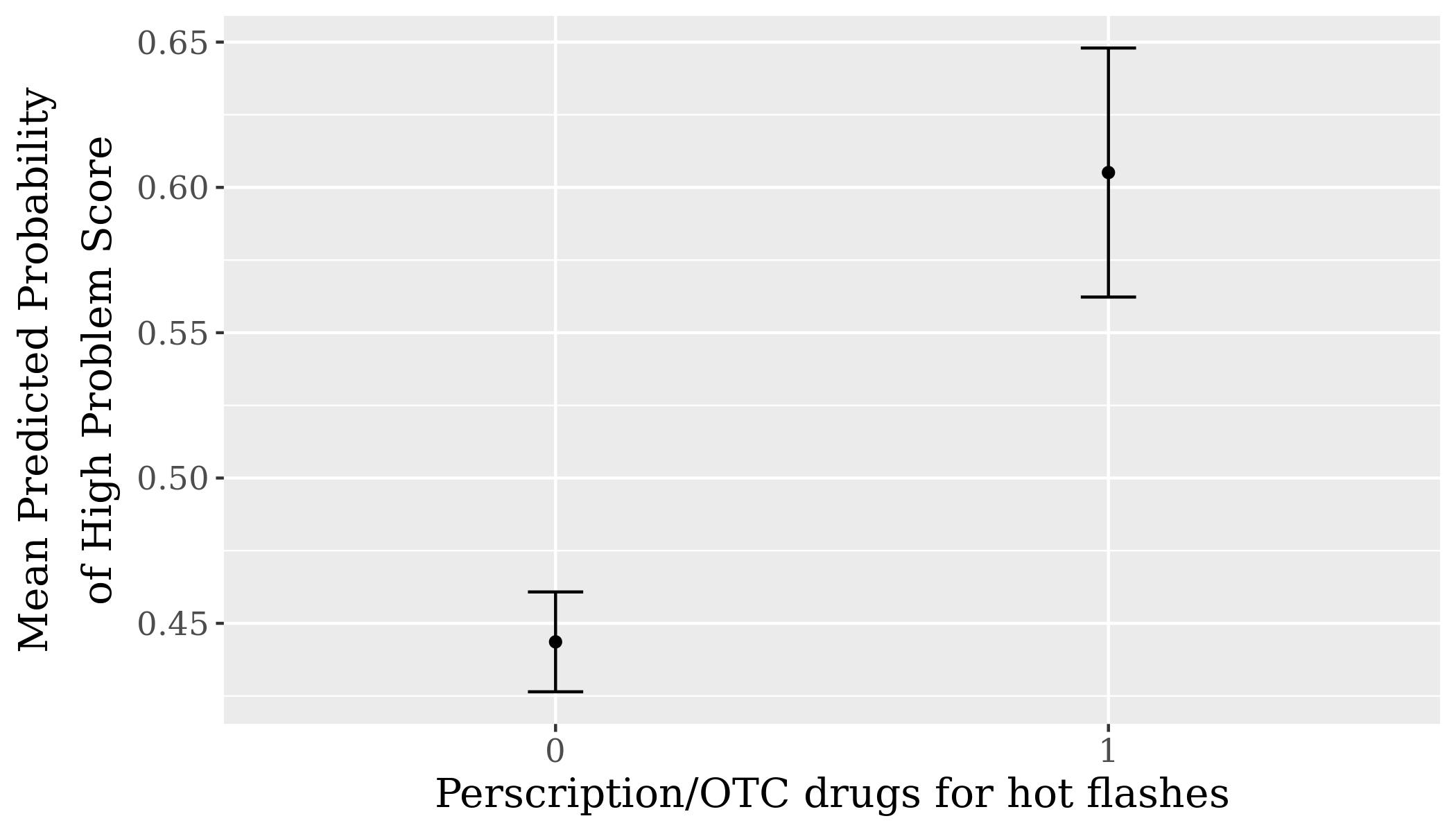

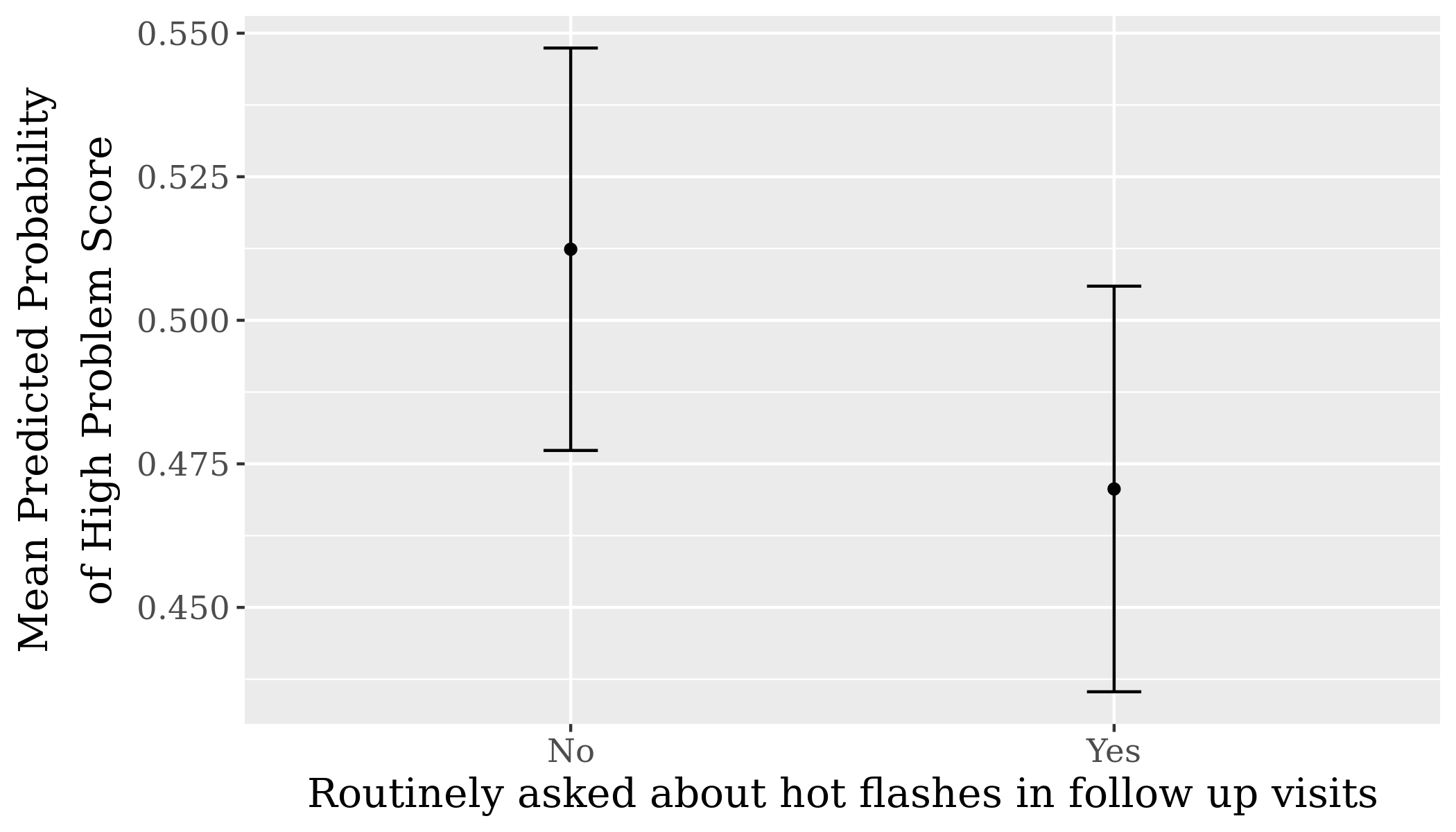


(a) (b)

**Figure B.1** The probability of hot flashes being a problem based on (a) whether the patient was prescribed or tried a drug to mitigate symptoms (b) whether the patient is routinely asked about VMS in follow up visits


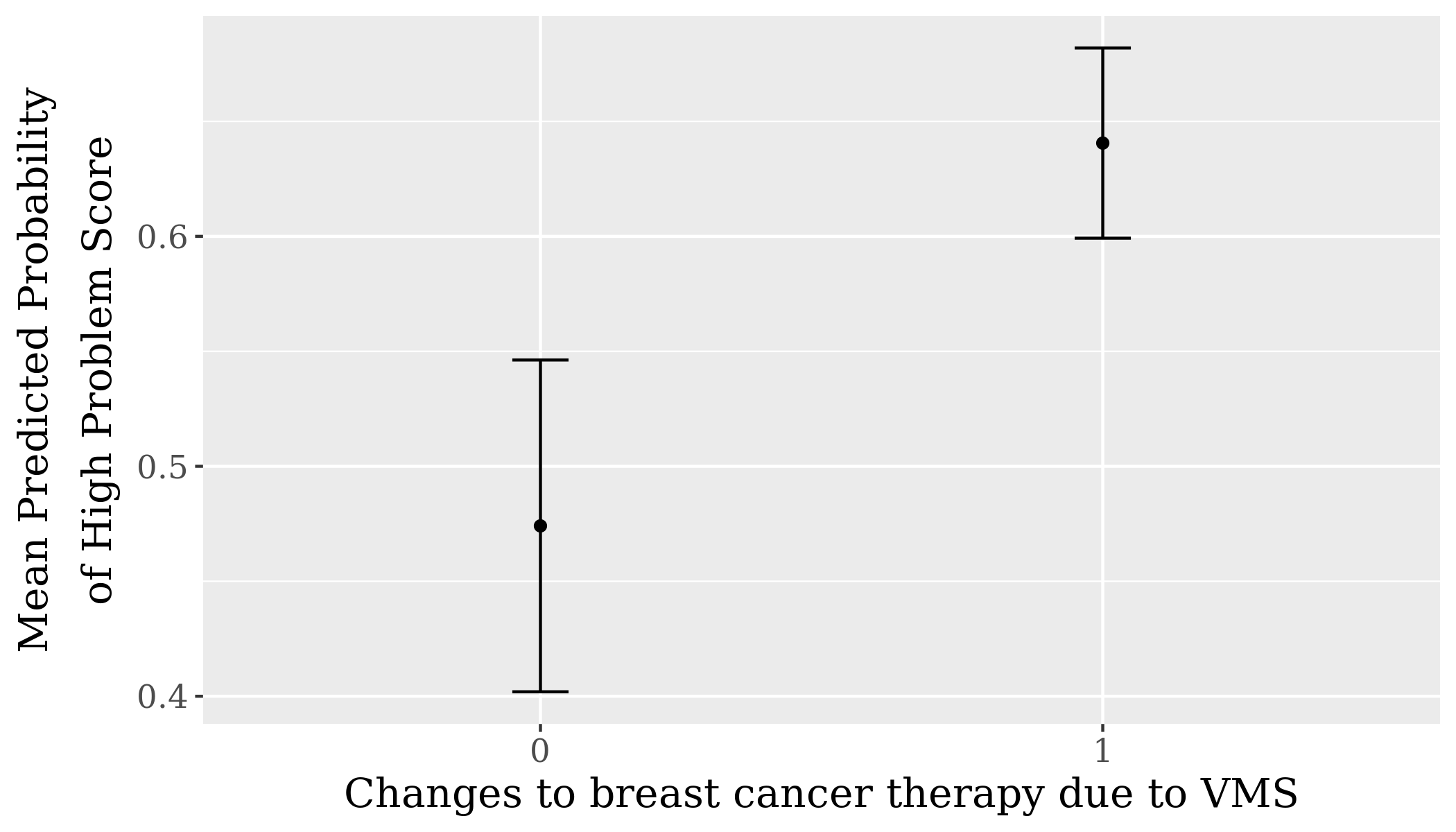


**Figure B.2** The probability of severe hot flashes based on whether a patient had a change in breast cancer treatment due to VMS


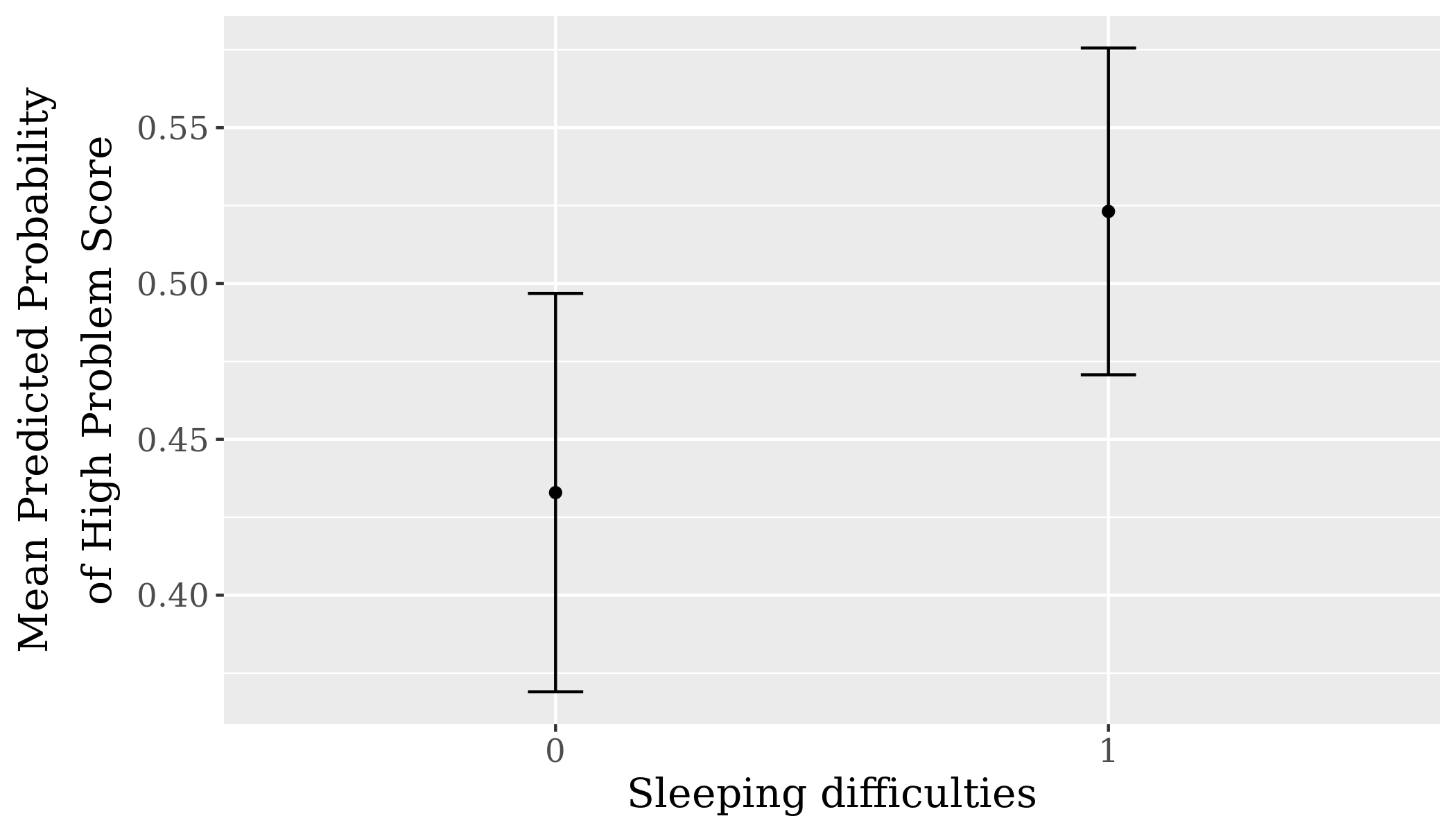

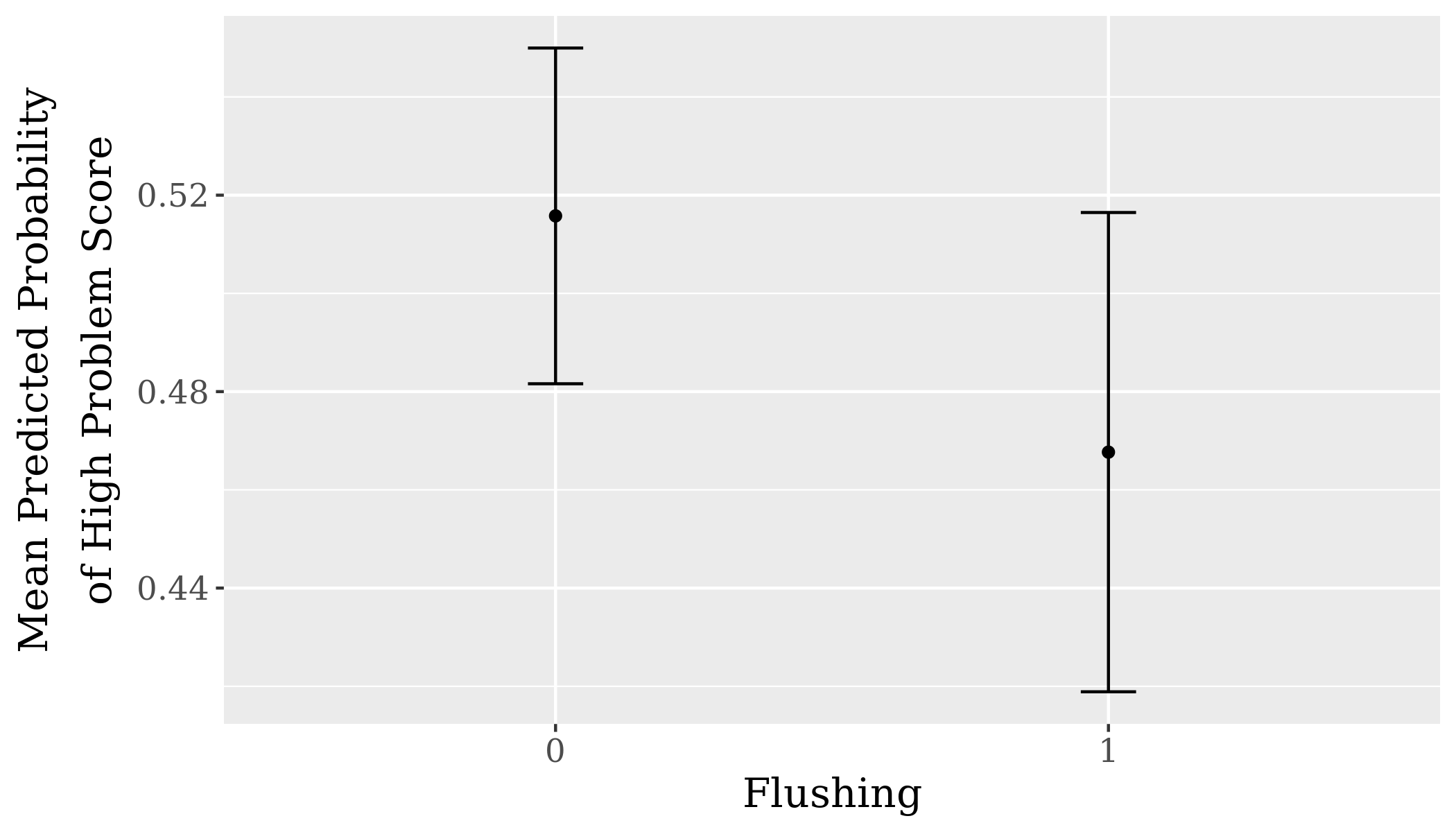


**Figure B.3** The impact of the top-rated bothersome symptoms on severity of VMS
