# Supplementary material for: Using machine learning to predict individual patient toxicities from cancer treatments
Source: Support Care Cancer. 2022 May 25;30(9):7397–406. doi: 10.1007/s00520-022-07156-6 (PMC9385785; doi:10.1007/s00520-022-07156-6)
Supplement: Supplementary file 3 — Supplementary file3 (DOCX 103 KB) [file 520_2022_7156_MOESM3_ESM.docx]

**Appendix C**

**Using Machine Learning to Predict Individual Patient Toxicities from Cancer Treatments**

Katherine Marie Cole ^a^, Mark Clemons^a,b^, Sharon McGee^a^, Mashari Alzahrani^a^, Gail Larocque^c^, Fiona MacDonald^c^, Michelle Lui^b^, Gregory R. Pond^d^, Lucy Mosquera^e^, Lisa Vandermeer^b^, Brian Hutton^f^, Ardelle Piper^g^, Ricardo Fernandes^h^, Khaled El Emam*^e,i^

a.) The University of Ottawa, Department of Medicine, Division of Medical Oncology. 75 Laurier Ave. E, Ottawa, ON, Canada. K1N6N5.

b.) The Ottawa Hospital Research Institute, Cancer Therapeutics Program, Ottawa, Ontario, Canada.

c.) The Ottawa Hospital Cancer Centre. 501 Smyth Road. Ottawa, Ontario, Canada. K1H8L6

d.) McMaster University, Department of Oncology, Hamilton, Ontario, Canada.

e.) CHEO Research Institute, University of Ottawa, Ottawa, Ontario, Canada

f.) The Ottawa Hospital Research Institute, Clinical Epidemiology Program, Ottawa, Ontario, Canada.

g.) University of Ottawa Health Services, Ottawa, Ontario, Canada.

h.) Division of Medical Oncology, Department of Oncology, Schulich School of Medicine & Dentistry, Western University, London, Ontario, Canada.

i.) University of Ottawa, School of Epidemiology and Public Health, University of Ottawa, Ottawa, Ontario, Canada.

**Correspondence:** Khaled El Emam

CHEO Research Institute, 401 Smyth Road, Ottawa, Ontario K1H 8L1

Fax:

Tel: 613-797-5412

Email: [kelemam@cheo.on.ca](mailto:kelemam@cheo.on.ca)

**Justifications for modeling approach**

The modeling approach we used was gradient boosted decision trees (GBDTs). There is evidence that across multiple analysis scenarios, boosted trees perform better than linear models, such as logistic regression [1]–[5]. Other strengths of GBDTs include 1) their ability to model complex interactions that do not need to be specified *a priori*, which is more important in an exploratory analysis than in a confirmatory context, and 2) improved handling of missing values. GBDTs also have been found to perform better than deep learning models on tabular datasets [6].

To estimate the true generalization error of the classifier if the training algorithm was applied on the full dataset, we use nested cross-validation (CV) [7]–[9]. It has been shown that this provides almost unbiased performance estimates [8].

We had an inner loop with 5-fold CV that is used for hyperparameter tuning, and a 10-fold CV in the outer loop for estimation of the generalization error. Previous studies found that different fold values for the two loops yielded comparable results.[9].

**Testing the normality assumption**

In computing the integer value for the cut-off we assumed that the HFNS Problem Rating Score was normally distributed for women with problematic and severe VMS. We test that assumption here.

Previous studies recruited women with problematic or severe VMS, whereas our study recruited women covering the full scale of severity, including those with low severity values on the HFNS Problem Rating Score. We can construct a simulated distribution for the two groups (high severity and low severity) and use that to test our assumption.


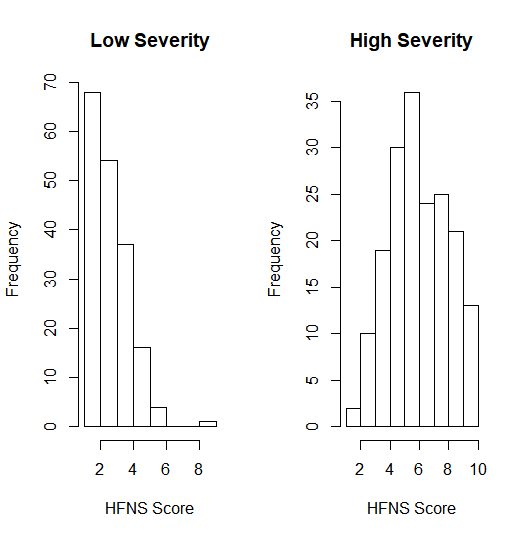


**Figure C.1:** The simulated low and high severity VMS distributions

From the studies cited in the main body of the paper, the mean HFNS score ranged from 5.88 to 6.3 (SD 2.2-2.6). We assumed that this followed a truncated normal distribution with a mean of 6.1 and SD of 2.4 (which are the mid-points from the range in the literature). The truncation is necessary since the HFNS score is bounded. This represents women with problematic or severe VMS. A single draw of 180 observations from that distribution is illustrated in Figure C.1.

Women with low severity VMS were assumed to follow a truncated normal distribution with a mean of 1.85 and SD of 1.85. This means that the average plus 1 SD for the low severity group coincides with the average minus 1 SD for the high severity group. The mean of the distribution for this group is the midpoint between the minimum and the cut-off point that separates the two distributions. A single draw of 180 observations from that distribution is illustrated in Figure C.1.


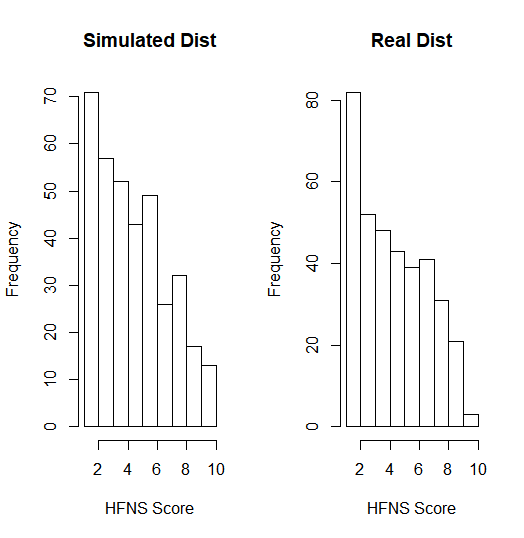


**Figure C.2:** The distributions for the simulated observations vs the real observations

We sampled 180 women from the low severity distribution and 180 women from the high severity distribution to create a 360 simulated HFNS scores that cover the full spectrum of VMS severity. This simulated distribution has parameters that are consistent with observations from the literature for high severity cases, and captures our assumptions of normality. This can be compared to the true distribution as illustrated in Figure C.2. The Kolmogorov-Smirnov (KS) test comparing the simulated distribution to the real observations from our survey had a p=0.2263, indicating that the real dataset does follow that simulated distribution. The conclusions do not change from repeated draws of the simulated distribution.

**Detailed model performance results**

The confusion matrix for the full model is shown in **Figure C.3** with additional performance metrics computed on it given in **Table C.1.**

We also constructed the model with the most important variables, making it easier to apply the model in practice because it has fewer variables that need to be collected. In principle a model with only the most important variables should give us the same performance as the full model. We will call this the “focused model”.

To select the top variables, we constructed a classification tree using the CART algorithm [10]. The advantage of classification trees is that they are more easily interpretable (they are not “black boxes”) and can be used for variable selection. These have the advantage over linear models in that we do not need make linearity assumptions and do not need to pre-specify the functional form of relationships and interactions. The variables selected in the final tree reflect the most important variables in the dataset. For this tree we used the gini-index for splitting 10-CV to compute the optimal complexity parameter for pruning. The decision tree constructed is shown in **Figure C.4.** The variables selected are the six top variables from the importance ranking that was computed from the GDBT model as show in in the main body of the paper.

The confusion matrix for the focused model is shown in **Figure C.5** and the performance metrics in **Table C.1.** As can be seen, the focused model has very similar performance results as the full model and has significantly fewer variables.

|  |  | PREDICTED | |
| --- | --- | --- | --- |
| ACTUAL |  | HIGH | LOW |
|  | HIGH | 116 | 62 |
|  | LOW | 54 | 128 |

**Figure C.3:** Confusion matrix for the full model


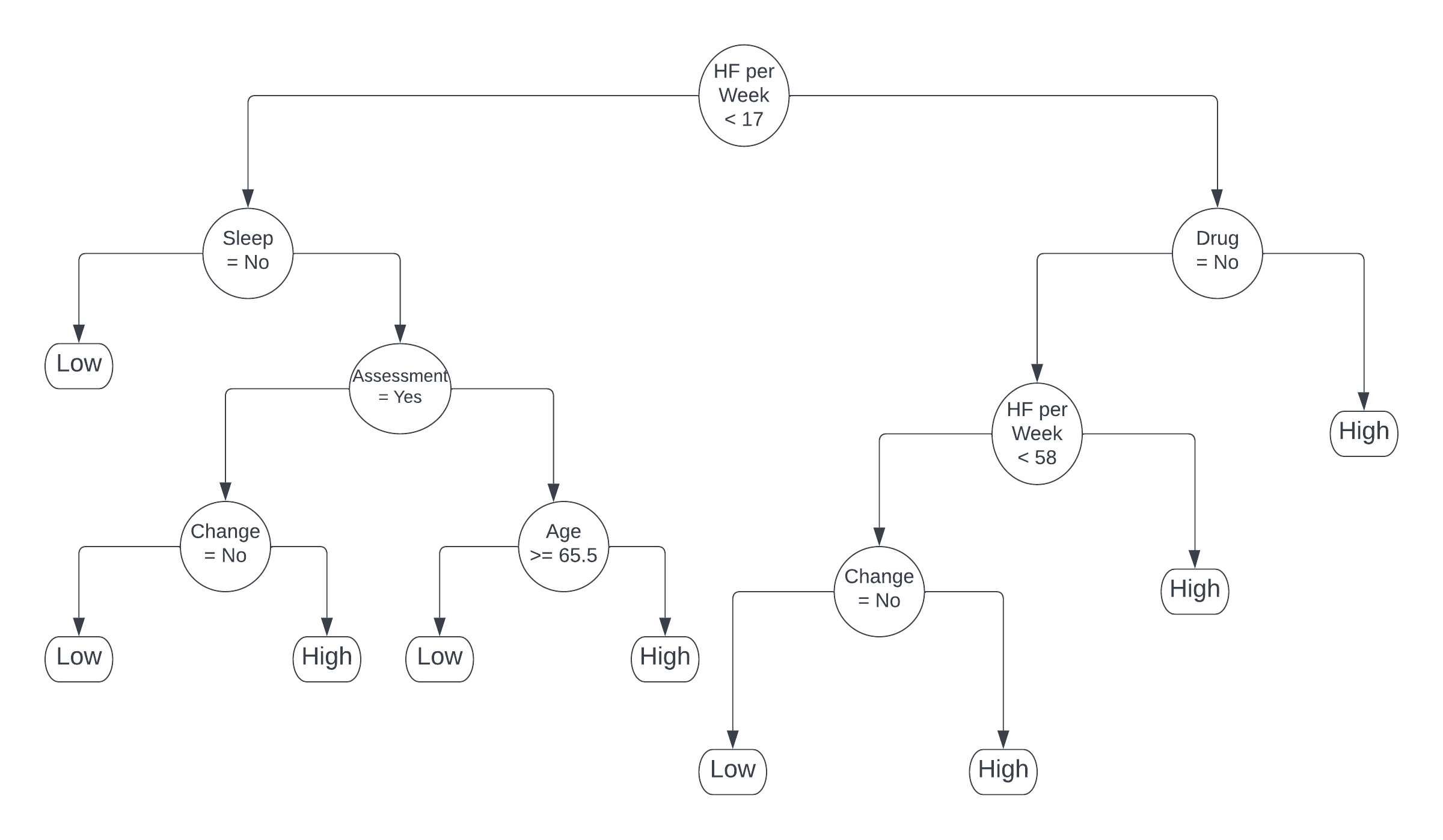


**Figure C.4:** The classification tree built on the full dataset. For each non-terminal node the left branch is the path taken if the condition is true

|  |  | PREDICTED | |
| --- | --- | --- | --- |
| ACTUAL |  | HIGH | LOW |
|  | HIGH | 117 | 61 |
|  | LOW | 57 | 125 |

**Figure C.5:** Confusion matrix for the focused model

| **Metric** | **Full Model** | **Focused Model** |
| --- | --- | --- |
| AUROC | 0.731 | 0.732 |
| AUPRC | 0.687 | 0.674 |
| Accuracy | 0.678 | 0.672 |
| F1 Score | 0.687 | 0.675 |
| Precision | 0.703 | 0.686 |
| Recall | 0.678 | 0.675 |
| Specificity | 0.687 | 0.684 |

**Table C.1:** Additional performance metrics of the full and focused models

**Removal of non-causal variables**

There were two variables in the full model that were affected by the cross-sectional nature of the survey. “Taking drugs to mitigate VMS” and “changes to BC treatment” were indicators of severe hot flashes, and thus are likely a result of problematic hot flashes, rather than early predictors of severe VMS.

We tested the model performance if we take these two variables out of the full model. The confusion matrix is shown in **Figure C.6** and the performance metrics are shown in **Table C.2.** The performance drop from the full model, demonstrates that these are important covariates that indicate problematic hot flashes.

|  |  | PREDICTED | |
| --- | --- | --- | --- |
| ACTUAL |  | HIGH | LOW |
|  | HIGH | 111 | 67 |
|  | LOW | 76 | 106 |

**Figure C.6:** Confusion matrix for the full model minus two variables: taking drugs to mitigate VMS and changes in BC treatment

| **Metric** | **Full Model minus two variables** |
| --- | --- |
| AUROC | 0.65 |
| AUPRC | 0.61 |
| Accuracy | 0.60 |
| F1 Score | 0.60 |
| Precision | 0.58 |
| Recall | 0.61 |
| Specificity | 0.60 |

**Table C.2:** Additional performance metrics of the full model minus two variables: taking drugs to mitigate VMS and changes in BC treatment

**References**

[1] A. Rousset *et al.*, “Can machine learning bring cardiovascular risk assessment to the next level?,” *European Heart Journal - Digital Health*, no. ztab093, Nov. 2021, doi: 10.1093/ehjdh/ztab093.

[2] S. F. Weng, J. Reps, J. Kai, J. M. Garibaldi, and N. Qureshi, “Can machine-learning improve cardiovascular risk prediction using routine clinical data?,” *PLOS ONE*, vol. 12, no. 4, p. e0174944, Apr. 2017, doi: 10.1371/journal.pone.0174944.

[3] R. K. Akyea, N. Qureshi, J. Kai, and S. F. Weng, “Performance and clinical utility of supervised machine-learning approaches in detecting familial hypercholesterolaemia in primary care,” *npj Digit. Med.*, vol. 3, no. 1, pp. 1–9, Oct. 2020, doi: 10.1038/s41746-020-00349-5.

[4] R. J. Desai, S. V. Wang, M. Vaduganathan, T. Evers, and S. Schneeweiss, “Comparison of Machine Learning Methods With Traditional Models for Use of Administrative Claims With Electronic Medical Records to Predict Heart Failure Outcomes,” *JAMA Network Open*, vol. 3, no. 1, p. e1918962, Jan. 2020, doi: 10.1001/jamanetworkopen.2019.18962.

[5] Y. Li, L. Jiang, J. He, K. Jia, Y. Peng, and M. Chen, “Machine Learning to Predict the 1-Year Mortality Rate After Acute Anterior Myocardial Infarction in Chinese Patients,” *TCRM*, vol. 16, pp. 1–6, Jan. 2020, doi: 10.2147/TCRM.S236498.

[6] R. Shwartz-Ziv and A. Armon, “Tabular data: Deep learning is not all you need,” *Information Fusion*, vol. 81, pp. 84–90, May 2022, doi: 10.1016/j.inffus.2021.11.011.

[7] M. Stone, “Cross-Validatory Choice and Assessment of Statistical Predictions,” *Journal of the Royal Statistical Society. Series B (Methodological)*, vol. 36, no. 2, pp. 111–147, 1974.

[8] S. Varma and R. Simon, “Bias in error estimation when using cross-validation for model selection,” *BMC Bioinformatics*, vol. 7, no. 1, p. 91, Feb. 2006, doi: 10.1186/1471-2105-7-91.

[9] D. Krstajic, L. J. Buturovic, D. E. Leahy, and S. Thomas, “Cross-validation pitfalls when selecting and assessing regression and classification models,” *Journal of Cheminformatics*, vol. 6, no. 1, p. 10, Mar. 2014, doi: 10.1186/1758-2946-6-10.

[10] L. Breiman, J. Friedman, C. Stone, and R. Olshen, *Classification and regression trees*, 1. CRC Press repr. Boca Raton, Fla.: Chapman & Hall/CRC, 1984.
